# The Effects of Photosensitizing Dyes Fagopyrin and Hypericin on Planktonic Growth and Multicellular Life in Budding Yeast

**DOI:** 10.3390/molecules26164708

**Published:** 2021-08-04

**Authors:** Oksana Sytar, Konstantia Kotta, Dimitrios Valasiadis, Anatoliy Kosyan, Marian Brestic, Venetia Koidou, Eleftheria Papadopoulou, Maria Kroustalaki, Christina Emmanouilidou, Alexandros Pashalidis, Ilias Avdikos, Zoe Hilioti

**Affiliations:** 1 Educational and Scientific Center “Institute of Biology and Medicine”, Department of Plant Biology, Taras Shevchenko National University of Kyiv, Volodymyrskya str., 64, 01033 Kyiv, Ukraine; Oksana.sytar@gmail.com (O.S.); a_kosyan@ukr.net (A.K.); 2 Department of Plant Physiology, Slovak University of Agriculture in Nitra, A. Hlinku 2, 949 01 Nitra, Slovakia; marian.brestic@uniag.sk; 3 Institute of Applied Biosciences, Centre for Research & Technology Hellas, 6th km Charilaou-Thermi Road, 57001 Thessaloniki, Greece; ntina_kotta@yahoo.com (K.K.); dimitrisvala@gmail.com (D.V.); vkoidoup@gmail.com (V.K.); eleftheria.papado93@gmail.com (E.P.); mari.krou@gmail.com (M.K.); xristina_emm@hotmail.com (C.E.); alex.pashalidis@gmail.com (A.P.); avdikos.elias@gmail.com (I.A.)

**Keywords:** fagopyrin, hypericin, yeast, morphogenesis, flow cytometry analysis

## Abstract

Naphthodianthrones such as fagopyrin and hypericin found mainly in buckwheat (*Fagopyrum* spp.) and St. John’s wort (SJW) (*Hypericum perforatum* L.) are natural photosensitizers inside the cell. The effect of photosensitizers was studied under dark conditions on growth, morphogenesis and induction of death in *Saccharomyces cerevisiae*. Fagopyrin and hypericin induced a biphasic and triphasic dose response in cellular growth, respectively, over a 10-fold concentration change. In fagopyrin-treated cells, disruptions in the normal cell cycle progression were evident by microscopy. DAPI staining revealed several cells that underwent premature mitosis without budding, a striking morphological abnormality. Flow Cytometric (FC) analysis using a concentration of 100 µM showed reduced cell viability by 41% in fagopyrin-treated cells and by 15% in hypericin-treated cells. FC revealed the development of a secondary population of G1 cells in photosensitizer-treated cultures characterized by small size and dense structures. Further, we show that fagopyrin and the closely related hypericin altered the shape and the associated fluorescence of biofilm-like structures. Colonies grown on solid medium containing photosensitizer had restricted growth, while cell-to-cell adherence within the colony was also affected. In conclusion, the photosensitizers under dark conditions affected culture growth, caused toxicity, and disrupted multicellular growth, albeit with different efficiencies.

## 1. Introduction

Many fungal pathogens can infect both plant and human hosts, and the baker’s yeast *S. cerevisiae* is offering a model system to study disease mechanisms and ways to control pathogenicity. The incidents of fungal infections caused by yeast species have increased lately, especially in hospitals. The factors that most frequently predispose humans to invasive fungal infection in hospitals are alterations in skin and mucous membranes (i.e., surgery), renal insufficiency, parenteral nutrition, therapy with corticosteroids or broad-spectrum antibiotics [1] and implanted prosthetic devices (catheters, dentures, heart valves) [2]. The currently available antifungal drugs like amphotericins, azoles and echinocandins are only fungistatic and not fungitoxic. Since their mechanism of inhibition is not specific, the human cells are also negatively affected by the drugs.

In nature, *S. cerevisiae* strains can be found on ripe fruits and are widely used in the fermentations of wine, beer, and other alcoholic beverages as well as in the baking industries. In addition, it was reported that certain strains of *S. cerevisiae* could penetrate the grapevine (*Vitis vinifera*) plants and delay plantlets growth, or even cause their death. This novel parasitic behavior was correlated to endopolygalacturonase activities and pseudohyphae formation [3]. Fungal pathogenic infections cause significant losses to the agricultural industry worldwide, as they account for 30 percent of emerging infectious diseases in plants.

Due to the ineffectiveness of current limited antifungals and emergence of resistance mechanisms, continued investigation on new antifungal agents with new modes of actions that minimize the side effects on host and the environment are needed. Natural products, especially those produced by plants, offer an immediate reservoir for drug discovery. Microbial exposure to photosensitizer molecules has been proposed as a potential alternative antifungal treatment (reviewed in [4]). Photosensitizers are natural dyes extracted from various plant tissues that can act as light-sensitive molecules. Cellular uptake of photosensitizers by either passive diffusion or active uptake followed by visible light energy can cause the excitation of the photosensitizer. Depending upon the intracellular distribution of the photosensitizer to organelles and other subcellular structures, their light-dependent excitation may upregulate the production of singlet oxygen and other reactive oxygen species (ROS), which may interfere with local vital functions and induce cell death.

*H. perforatum* L. (St. John’s wort), an herbaceous perennial plant native of Europe, has been considered a medicinal plant for over 2000 years. The ancient Greek physicians Galen, Dioscorides, Pliny, and Hippocrates prescribed it as a diuretic, antimicrobial for wound healing, treatment for menstrual disorders, and cure for gastrointestinal problems and snakebites [5,6,7]. Hypericin, a major active constituent of SJW flowers, is a naphthodianthrone. The pigment is responsible for the red color of SJW oil and is highly photoreactive when exposed to fluorescent light (excitation spectra 650 nm at 295 K and 620 nm at 77 K) [8] due to the presence of two closely located oxygen atoms (approximately 2.5 Å apart), which allow a hydroxyl hydrogen to be in constant flux between the two oxygen atoms [9]. Hypericin is well known for its photodynamic action that can induce photosensitization in cells and cytotoxicity [10,11,12]. In the presence of oxygen and light, hypericin leads to the upregulated production of intracellular reactive oxygen species (ROS), which may also cause cytotoxicity in grazing animals who eat SJW weed. However, several in vitro studies with different experimental systems and/or setups support cytotoxic or antiproliferative action of hypericin, even in the dark [13,14,15,16,17,18].

Fagopyrin, a red pigment, is a closely related molecule to hypericin. It contains a naphthodianthrone skeleton with two molecules of piperidines and has photosensitizing properties. The compound is found in *F. esculentum*, commonly known as buckwheat, which is consumed worldwide as a “functional food” [19,20], particularly due to their high-quality protein, abundant phenolic compounds and balanced essential amino acids and minerals [21,22]. Hypericin has intense absorption spectrum in the visible region with a maximum absorption peak of about 599 nm [23], low photo bleaching, short half-life (27 h even at a dosage of 1500 μg/kg), and a wide excitation range [24,25].

The aim of this study was to examine the fungistatic and fungitoxic effects of fagopyrin and hypericin on baker’s yeast planktonic and multicellular growth under dark conditions, which are relatively understudied. Dark conditions do not require illumination devices of a specific wavelength, are not limited to surface treatments and topical applications, and are independent of the penetration depth of visible light [26]. The non-light-activated fagopyrin and hypericin interfered with cell cycle progression, induced death, and altered the morphology and fluorescence state of cells, suggesting their potential use as antifungal agents.

## 2. Results and Discussion

### 2.1. Photosensitizers Disrupt Cell Cycle Progression and Morphogenesis

**Figure 1:**
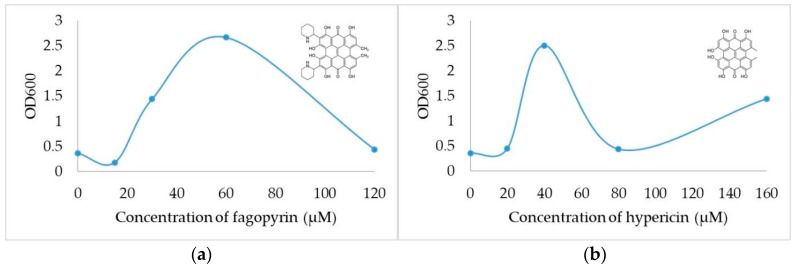
Representative dose–response curves of photosensitizers on yeast growth. Cultures in suspensions with identical initial density (OD600) were allowed to grow for 3 h (doubling time 1.5 h) after addition of photosensitizers: (**a**) Effect of fagopyrin (C40H34N2O8); (**b**) Effect of hypericin (C30H16O8). Of note, the similarity and the differences of the two structures: both compounds contain a naphthodianthrone skeleton, but fagopyrin has two piperidines, which are not present in hypericin [29].

Photosensitizing dyes upon uptake by the cells and transport to cellular components passively or by membrane-associated translocation processes including endocytosis [27] have the potential to influence vital functions. Antibacterial photodynamic inactivation of fagopyrin from the *Fagopyrum tataricum* flower against Streptococcus mutans and its biofilm was determined for the first time by Kim et al. 2021 [28]. Less is known about the biological activities of photosensitizers under dark conditions (without irradiation), which are not based on the combined action of the photosensitizer, light and molecular oxygen, in suppressing the growth and development of fungi. Budding yeast as a single-celled organism with morphological markers that discriminate cell cycle phases is an ideal model system to study the effect of natural photosensitizing dyes like fagopyrin and hypericin on cell cycle progression. The morphological markers used in this study were the presence and size of a bud. To gain deeper insights into the dark effects of fagopyrin and hypericin, the relationship between the dose of the photosensitizer and the biological response that it elicits was studied by monitoring culture growth for 3 h following increasing concentrations of the photosensitizers in YPD medium. Dimethyl sulfoxide (DMSO), a useful solvent in pharmacology and toxicology that dissolves both polar and non-polar molecules, was used as a negative control in our yeast growth experiments since it was the solvent used to solubilize the fagopyrin and hypericin extracts. DMSO is generally nontoxic below 10% (*v*/*v*) and in our experiments a 2% (*v*/*v*) was used as control. As it can be seen in Figure 1, fagopyrin and hypericin dose–response curves show different trends between prevailing-growth and prevailing-decline and the overall shape of the dose–response curve, and the photosensitizer concentration-dependence. Fagopyrin treatment triggered a biphasic dose–response curve with a delayed onset of growth stimulation at low doses (15 to 60 µM), maximal attainable response at 60 µM and a slow decline (shallow slope) in growth up to 120 µM) (Figure 1a). On the contrary, hypericin treatment led to a triphasic dose–response curve with an initial lag phase, a rapid growth (steep slope) stimulation from 20 to 40 µM, a maximal attainable response at 40 µM, a fast decline up to 80 µM, followed by a slow adaptation (trough) up to 100 µM and a slow regrowth phase (Figure 1b). The different slopes observed in the growth responses for the two photosensitizers indicate different potencies, possibly due to differences in the absorption and elimination rates. Notably, yeast cells scatter light rather than absorb it, like soluble molecules do, and so they do not follow the Lambert–Beer Law, which states that there is a logarithmic relationship between the transmission of light through a sample. In yeast culture, the optical density (OD600) readings from light scattering are attributed not only to the number of cells but also to the size (delays in cell cycle progression) and shape of cells, the presence of dead cells and cell debris, as well as the formation of cell groups (nondividing cells).

**Figure 2:**
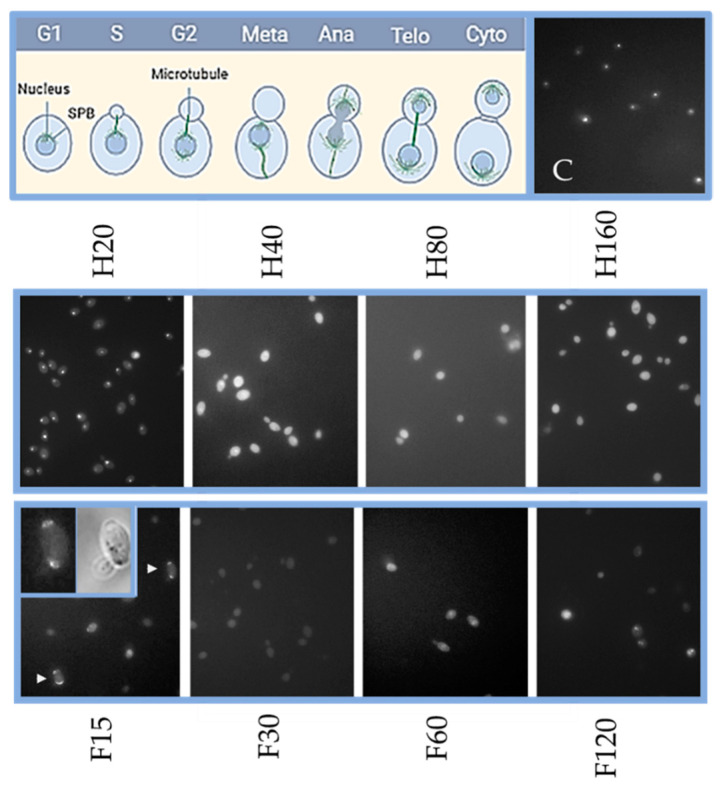
Dose-dependent effects on cell cycle by photosensitizers. Representative images from asynchronous log-phase cells grown for 3 h in the absence (C) or presence of fagopyrin (F) and hypericin (H). A schematic figure depicting the landmark events of the normal cell cycle progression (top left panel). Cells were stained with the fluorescent stain DAPI to visualize nuclei. DAPI staining revealed that exposure to photosensitizers caused diffuse DAPI signal in a dose-dependent manner. Arrowheads (white) show cells that underwent precocious mitosis in the absence of bud. A more detailed view of nuclei and cell morphology (bright field) is in the insets.

To understand the growth-inhibitory effects of the photosensitizers in the dark, microscopic evidence from cell morphology landmark events (bud emergence, cell separation) was combined with nuclear behavior based on fluorescence images of 4′-6-diamidino-2-phenylindole (DAPI)-stained nuclei (Figure 2). The DAPI-based analysis relies on the stoichiometric binding of the DAPI to DNA, thus enabling the detection of content variation occurring throughout cell cycle phases. Based on DAPI staining, we were able to detect an unexpected finding that fagopyrin at 15 µM induced DNA duplication, a characteristic of the S phase, in cells that appeared elongated but lacked a bud. Thus, fagopyrin at this level caused anomaly in cell morphogenesis and raised difficulty in validating the DAPI-stained nuclei data. Other studies have reported nuclear division in conditional mutants, which were defective in bud formation (reviewed in [30]), shedding light on the genetic regulation of the spatiotemporal relationship of nuclear and cytoskeletal dynamics during the cell division cycle. Under normal conditions, morphogenetic events of the cell division cycle, such as initiation of bud emergence in late G1 and bud growth in S through G2 phase, are preceded by the localization of actin cytoskeleton and other elements and are in synchrony with the nuclear events. Further, using fluorescence microscopy for DAPI staining, we observed brightly stained nuclei with increasing concentrations of photosensitizers, an indication that yeast cells are dying (or have already died) by apoptosis since chromatin condensation intensifies the DAPI staining (Figure 2, panels H40, H80, H160, F60, F120).

**Figure 3:**
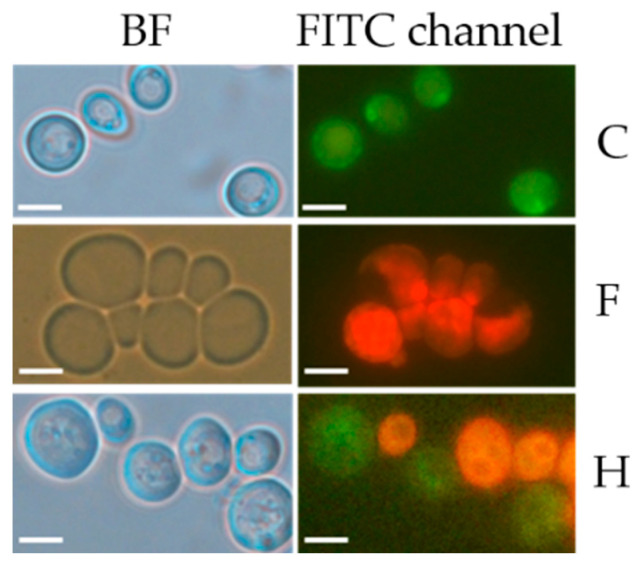
The effects of fagopyrin and hypericin on yeast cells. Yeast cells imaged by fluorescence microscopy in FITC channel using 488 nm laser excitation in cultures with no phytochemicals (C) and in the presence of 100 µM fagopyrin (F) or 100 µM hypericin (H) for 3 h. Cell showing natural green autofluorescence (C), red fluorescence under fagopyrin exposure (F) and a mixture of green (natural autofluorescence) and red fluorescence depending on cell cycle stage under hypericin exposure. After the addition of fagopyrin, cells arrested in mitosis cannot divide, whereas cells exposed to hypericin also have division defects and accumulate as large-budded cells. The same cells imaged in the bright field (BF). Microscopic images taken with 100× objective. Scale bar, 5 µm.

To further characterize the effects of high (100 µM) fagopyrin or hypericin concentration in yeast, we used microscopy to visualize the cells. Thus, it became evident that, at this concentration, the majority of the cells (approximately 81%) were in G1 phase, a moderate percentage in G2 (14.8%), and a low percentage in S (2.8%) and M (1.4%) phases. In addition, we observed aberrant cell morphologies arrested in mid anaphase (spindle and DNA mass through the neck of large-budded cells) in fagopyrin-treated cultures (Figure 3). Although nuclear division and cytokinesis did not occur and mother and daughter cells did not separate, they initiated buds resuming, thus, a new cell cycle. This resulted in the formation of chained cells (group of cells). Overall, single G1 cells and groups of chained cells were the most prominent phenotypes detected in fagopyrin treatment.

Similarly, hypericin treatment arrested several cells in mitosis but they had greater size compared to fagopyrin treatment, indicating that cells delayed cell cycle progression for a longer time (Figure 3). In the dark, hypericin inhibited DNA synthesis in DA-3 and SQ2 cell lines in a cytostatic rather than cytotoxic manner [13]. Under dark, hypericin induced retardation at G2-M phase in tumor cells, increased cell volume and multinucleation, leading to mitotic cell death. Hypericin-mediated inhibition of murine tumor cell growth in the dark was due to the inactivation of Hsp90 by enhanced ubiquitinylation, which resulted in the destabilization of Hsp90 client proteins [14]. Notably, other studies found that reduction of Hsp90 protein levels promotes aberrant cycling and premature mitosis [31,32]. Fluorescence microscopy revealed that the two lipid-soluble photosensitizers accumulated in the cell membranes, and they appeared quite stable in emitting a fluorescence signal when briefly excited. Changes in cellular shape and mitotic spindle positioning between mother and daughter cells occur during mitotic progression, and these events are orchestrated by the coordinated activities of cytoskeletal rearrangements (microtubules, actin and actin-binding proteins) and membrane dynamics. A failure to execute normal mitotic progression may induce de novo DNA damage during and after mitosis. Several interesting features of the photosensitizer treatments were noted, as multiple yeast cells exhibited incomplete nuclear division and/or cytokinesis, indicating a genotoxic effect that does not allow the normal cell cycle progression. Notably, in both treatments, cells continued to grow during the arrest, increasing their volume/surface ratio. Thus, fagopyrin and hypericin acted as micromolar inhibitors in a cell cycle-dependent manner. The different results of their effects suggest different defects in the underlying regulatory mechanisms of cell cycle progression.

In summary, our results highlight that the remarkable ability of the yeast cells to divide accurately, which is vital for genome stability, was perturbed in the presence of the two structurally related photosensitizers.

### 2.2. FC for Determination of Cell Viability and Apoptosis after Exposure to Photosensitizers in the Dark

**Figure 4:**
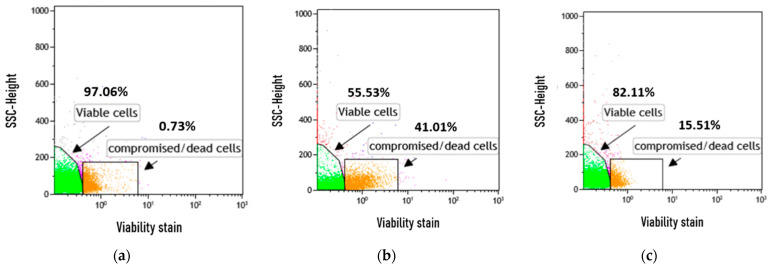
Flow cytometric analysis of photosensitizer-treated yeast cultures for discrimination of live from dead cells. Yeast cells were cultured in YPD medium treated with photosensitizers for 3 h and then stained with the BD Horizon Fixable Viability Stain (FVS 660). Representative FC plots of (**a**) untreated cells and treated with either (**b**) fagopyrin or (**c**) hypericin. The analysis of the in vitro assay showed that fagopyrin treatment resulted in a greater (41.01%) percentage of necrotic cells compared to hypericin treatment (15.51%).

The effects of photosensitizers on the growth of yeast cells in vitro were studied using FC. FC allows a large number of cells to be analyzed at once and provides real-time quantitative data. Analyses were performed using the amine-reactive fluorescent dye FVS 660 that discriminates viable from nonviable cells based on fluorescence intensity. Typically, live cells have intact membranes and are impermeable to the dye, while dead cells allow for the intracellular diffusion of the dye. The covalent binding of the dye to higher concentrations of amines in dead cells compared to living impermeable cells results in a higher level of fluorescence intensity. The cell viability assays following incubation with the photosensitizing dyes under dark conditions are shown in Figure 4a–c. As shown, in the untreated cell population, 82.01% were viable and 16.85% were compromised/dead. Incubation with fagopyrin increased the percentage of dead cells significantly up to 41.01% (Figure 4b), revealing that fagopyrin induces cytotoxicity at the 100 µM dose level. On the contrary, hypericin had reduced effect on membrane integrity/survival, as only 15.51% of cells died (Figure 4c), indicating that hypericin is not as toxic at the same concentration (100 μM). Previous yeast-based genotoxicity tests revealed that hypericin did not cause DNA alterations [16]. Our results clearly show that the two treatments had a differential effect on yeast planktonic growth, and flow cytometry can efficiently discriminate these responses and provide quantitative data.

**Figure 5:**
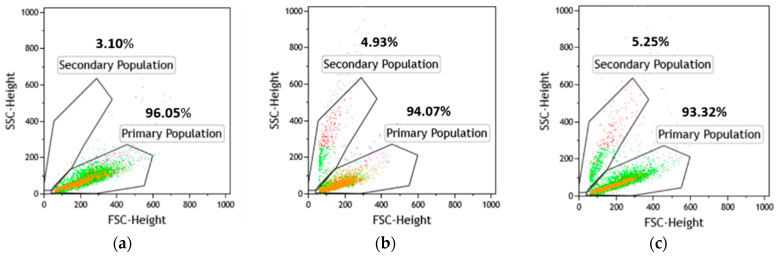
Distinction of morphological classes in unsynchronized yeast populations. Representative FC plots from (**a**) untreated cultures and cultures treated with (**b**) fagopyrin 100 µM, (**c**) hypericin 100 µM. Changes in light-scattering properties of cells exposed to photosensitizers. The primary populations in the treated culture represent cells that have similar light-scattering properties with the untreated population. The secondary populations have diminished forward scatter (FSC) and small cell size with dense structures (SSC).

In addition, FC analysis revealed that the photosensitizer treatments changed the morphology of the cells, while a new group of small cells with internal complexity (dense structures) emerged (Figure 5).

In summary, the toxicity of the two photosensitizers was not of the same order of magnitude since fagopyrin resulted in a 2.6-fold increase in toxicity compared to hypericin. The delay in cell cycle progression observed under photosensitizer treatment likely contributed to the increased number of dead cells.

### 2.3. Effect of Fagopyrin and Hypericin on Static Cultures

**Figure 6:**
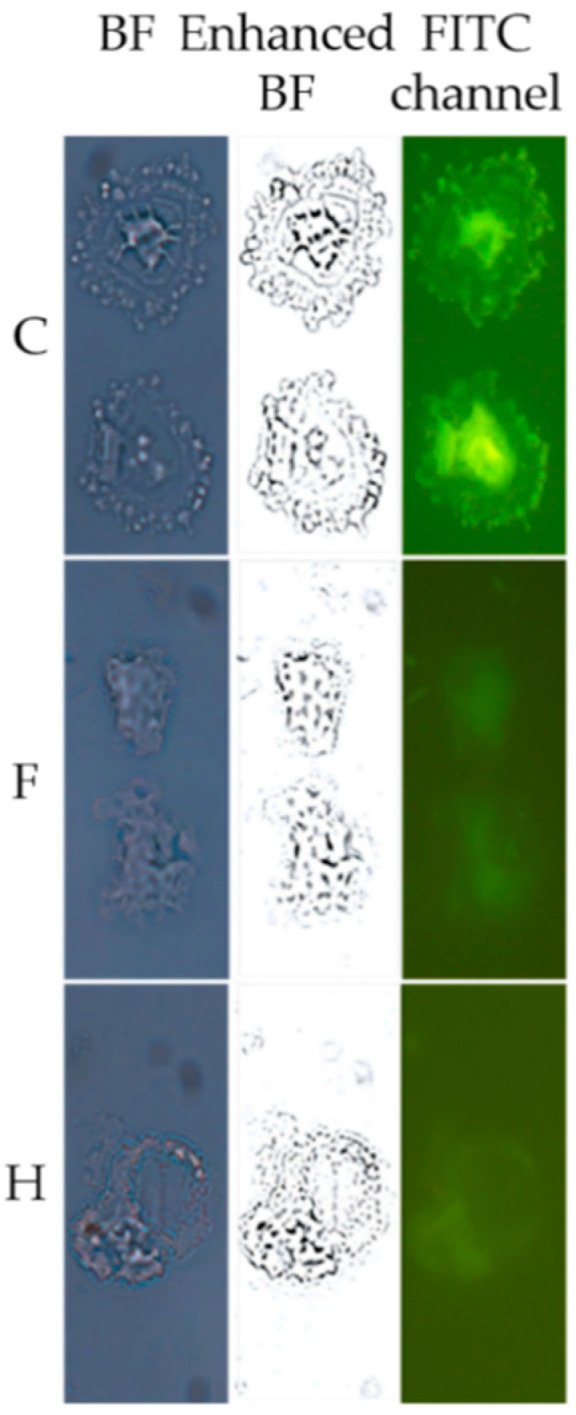
Alterations in biofilm morphology and fluorescence under fagopyrin and hypericin exposure. Planktonic growth for 24 h in YPD medium supplemented with fagopyrin (100 µM) (F), hypericin (100 µM) (H) or DMSO (C). The cells were imaged using bright-field (BF) microscopy and after laser excitation at 488 nm (FITC channel) with 100× objective.

To establish an infection within the host organism, yeast must survive hostile environments. An adaptive mechanism to stressful environments is the cell–cell adhesion of free-floating (planktonic) cells, known as “flocculation”, and formation of multicellular growth, like biofilms and colonies, which attach to surface. Sessile yeast exhibit a phenotype that is distinct from that of their planktonic counterparts. Specialized proteins mediate cell attachment, and these are critically important in allowing yeast cells to form multicellular growths. To evaluate fagopyrin and hypericin effect on yeast multicellular growth, we investigated cell morphologies after a 24 h treatment. Biofilm-like formation was detected in cultures treated with 100 µM of either fagopyrin or hypericin, which was different from the control culture (Figure 6). Under epifluorescence microscopy, the untreated control biofilms exhibited increased autofluorescence in the center, with little fluorescence in concentric cycles and the periphery of the biofilm. The majority of autofluorescence in UV-excited living cells originates from mitochondria, lysosomes [33] and different endogenous molecules, including tryptophan [34] and NAD(P)H, an indicator of the redox state of the cells [35,36]. Endogenous fluorophores are involved in structural and metabolic functions, structural functions as well as the phenotypic and physiological state of cells [37]. The distribution of fluorescence in yeast cell assemblages after brief UV exposure was analyzed (Figure 6). A direct comparison of the fluorescence signal from the control cells with the photosensitizer-treated cells showed that fluorescence was lost under photosensitizer treatment, suggesting changes in cell metabolic activity.

### 2.4. Effect of Photosensitizers on Colony Growth

Yeast cells growing on solid media execute different developmental programs compared to planktonic growth. The colony is a multicellular system of highly differentiated cells [38,39]. Assessment of the effects of photosensitizers uptake by *S. cerevisiae* was determined by examining colony growth on YPD plates containing either fagopyrin (F) or hypericin (H) and comparing them to control (C) plates. The presence of photosensitizers in the YPD medium did not cause a significant change (*p* > 0.05) in the colony diameter, perimeter and circularity, but the morphology of the colony was different since fagopyrin resulted in less-dense colonies based on light microscopy observations and hypericin in smooth borders that exhibited signals (Table 1).

**Figure 7:**
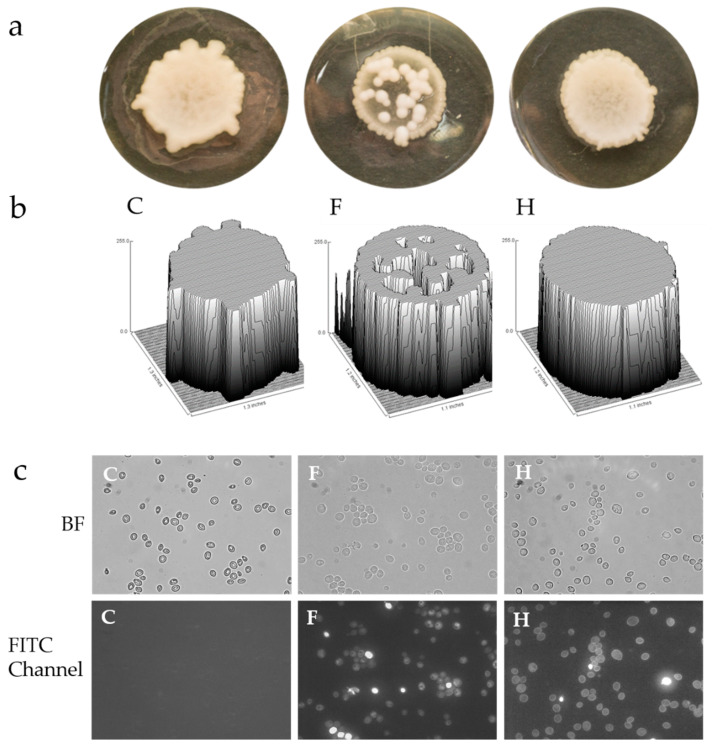
Photosensitizer effect on colony growth. Colonies of *S. cerevisiae* grown on solid-medium change morphology after exposure to either fagopyrin or hypericin. (**a**) Yeast cells were grown for 15 days in YPD plates without photosensitizers (C) and in media containing either 100 µM fagopyrin (F) or 100 µM hypericin (H). (**b**) Surface plots of the colony intensities of pixels in a grayscale using ImageJ. (**c**) Microscopic images of cells from the colonies in the bright field (BF) and with FITC channel (488 nm laser excitation) showing different fluorescence patterns for fagopyrin and hypericin. Arrowheads (white) show cells with aberrant morphologies. Scale bar, 15 μm.

We next examined under microscopy the morphology of the cells in the colonies. Control yeast cells exhibited low fluorescence signals and appeared well separated. On the contrary, fagopyrin or hypericin treatment (100 µM) caused a significant increase in fluorescence signal emitted by the photosensitizer and the cells appeared in a multicellular growth (Figure 7c, bottom middle and right panels). In the phytopathogenic oomycetes *Phytophthora citrophthora*, fagopyrin had a greater inhibitory effect on the radial colony growth compared to hypericin treatment, while detailed microscopic analysis revealed alterations in the hyphal shapes and fluorescence intensities after exposure to either fagopyrin or hypericin [40]. 

## 3. Materials and Methods

### 3.1. Isolation of Fagopyrin and Hypericin

Hypericin and fagopyrin were prepared and purified with silica and Sephadex LH-20 column chromatography as described previously [18]. For the cell culture experiments, hypericin and fagopyrin stocks were dissolved in sterile DMSO and stored at −20 °C under dark conditions.

### 3.2. Yeast Strain, Culture Growth and Dose–Response Studies

For all experiments, freshly growing cultures of the yeast strain BY4741 (*MAT*a; *his3*Δ1 *leu2*Δ0 *met15*Δ0 *ura3*Δ0) ([41]; EUROSCARF) grown in YPD medium (yeast extract 10 g/L, peptone 20 g/L, and dextrose 20 g/L) were used. For growth-inhibitory tests, yeast cultures at the mid-log phase were split in subcultures and each one was treated with or without the addition of photosensitizers. Different concentrations of the components were used for fagopyrin (0–120 µM) and hypericin (0–160 µM). The solvent DMSO was used at 2% in control cultures. The cells were left to grow at 30 °C for 3 h in the dark. Yeast culture density was measured by optical density at 600 nm (OD600) using a spectrophotometer (Quawell Q5000, San Jose, CA, USA).

### 3.3. Cell Nuclei Staining

For nuclei staining, cells cultured in YPD were harvested and washed with 1X PBS. Subsequently, cells were placed on a glass slide with a drop of mounting medium (StayBrite Hardset Mounting Medium, Biotium, Fremont, CA, USA) containing DAPI (1 µg/mL) and imaged by fluorescence microscopy on a Zeiss Axioscope 40 microscope (Carl Zeiss, Jena, Germany).

### 3.4. Biofilm Assays in Static Liquid Culture

Static biofilm assay was carried out using overnight yeast liquid cultures in Erlenmeyer flasks without shaking in the presence or absence of photosensitizers. Biofilms were imaged after 24 h using a Zeiss Axioscope 40 with 100× lenses. For morphology studies, bright-field imaging was carried out, and for epifluorescence imaging of the biofilms, excitation wavelengths of 345 nm, 495 nm, 595 nm and emission wavelengths of 455 nm, 519 nm, 613 nm, respectively, were used.

### 3.5. Effect of Photosensitizers during the Establishment of Biofilm

For the study of biofilm formation, a wild-type *S. cerevisiae* strain (BY4741) was inoculated on each YPD screening plate containing one of the following: DMSO solvent-only (2%, control), fagopyrin or hypericin. To ensure consistency of compound activity and data reproducibility, technical and biological replicates were performed in each experiment, and the experiment was replicated three times.

### 3.6. Colony Parameters

Colony features from digital images of petri dishes were analyzed using the open-source software ImageJ [42] with a ruler on the original image to set the scale by calibrating the area with a known distance. Several plugins in the ImageJ menu were used to open (File→Open) the colored file, highlight the region of interest using the circle tool to highlight the colony (Edit→Highlight), subtract the background (Edit→Clear outside), convert the colored image to 16-bit greyscale (Image type→16-bit), apply thresholding (Image adjust) on a black-and-white image and finally analyze the three-dimensional graph of the intensities of pixels in a grayscale image (Analyze→Surface plots). Features of the thresholded images were extracted for perimeter, diameter, and colony circularity. A value of 1 for circularity indicates a perfect circle. At least six images were analyzed, and the average values were used.

### 3.7. Statistical Analysis

Colony measurements were performed in 6 colonies and the experiments were repeated twice. Data are presented as means ± standard error of the mean. ANOVA analysis was carried out to find out the level of significance and Tukey’s post hoc test. *p*-values lower than 0.05 were considered significant. The statistical analyses were performed with the GraphPad Prism software (version 5).

### 3.8. Microscopy Studies

For examining cell cycle stage, 100 cells were counted from each treatment under light microscopy. Images for morphology analysis were captured using a Zeiss Axioscope 40 epifluorescence microscope, coupled with a ProgRes CF cool camera (Jenoptik, Jena, Germany), and ProgRes Mac CapturePro software (Jenoptik, Jena, Germany) and equipped with different objectives (EC Plan-Neofluar 100x oil, A-Plan 40x/0.65na Ph2 Var2, A-Plan 10x/0.2, A-Plan 5x/0.12), excitation and emission filters for DAPI (455 nm), FITC (519 nm) and Texas Red (613 nm) for detection of fluorescence. A bright-field image was acquired for each treatment to facilitate morphology identification.

### 3.9. Cell Viability Assay

Prior to FC viability assay, a series of preliminary experiments were performed in order to study the effect of different concentrations of DMSO on cell death with cells treated with DMSO only and to determine the optimal concentration of DMSO that does not cause cell death. Cell cultures were treated with the photosensitizers fagopyrin (100 µM) and hypericin (100 µM) for 3 h along with a nontreated control culture. A minimum of 10,000 events were acquired for discrimination of viable from nonviable yeast cells using a BD FC Calibur Flow Cytometer equipped with a blue and a red laser. Cell viability was assessed using the FVS 660 stain (BD Biosciences, Franklin Lakes, NJ, USA) for 10 min in cells previously washed with 1X PBS. At the end of incubation, the excess stain was washed off with 1X PBS. The cells were gated with autofluorescence according to forward scatter/side scatter; secondary gates were set based on cell viability. Data analysis was performed using the Flowing Software Version 2.5.

### 3.10. Determination of Cell Size and Internal Complexity by FC

The photosensitizers fagopyrin (100 µM) and hypericin (100 µM) were used for this analysis. An exponentially growing culture of BY4741 in YPD medium in the presence or absence (DMSO only) of each photosensitizer for 3 h was spun down and washed with phosphate-buffered saline (PBS). Subsequently, the cells were fixed in a final concentration of 70% ethanol at room temperature for 30 min, centrifuged, and the cell pellet was resuspended in PBS to rehydrate the cells. Analysis was performed in 10,000 cells per sample by a FACScan flow cytometer (Becton Dickinson, Erenbodegem-Aalst, Belgium) and aggregated cells were gated out. The analysis was performed in triplicate samples.

### 3.11. Software

Illustration was created with BioRender.com (27 April 2021).

## 4. Conclusions

Altogether, the present data showed that fagopyrin and hypericin uptake by the *S. cerevisiae* cells interfered with their biology both in planktonic and biofilm-like growth and accumulated in organelles and near the nucleus. A fraction of cells was unable to complete division at mitosis and initiated a new budding cycle ending up in the formation of cell aggregates. Based on FC analysis, fagopyrin caused greater toxicity to cells compared to hypericin during the 3 h exposure. Further, the autofluorescence pattern found in biofilm-like structures was severely affected in both liquid and solid medium and it was accompanied by abnormal morphologies. These findings suggest that the two photosensitizers have antimicrobial properties against unicellular and multicellular growth of yeast.

## Figures and Tables

**Table 1:** Effect of fagopyrin and hypericin on colony features. Quantitative results expressed as means ± standard error of the mean (n = 6) from representative colonies grown for 15 days in YPD plates under different treatments: control (C, no photosensitizer), 100 µM fagopyrin (F) and 100 µM hypericin (H).

Colony	Diameter (cm)	Perimeter (cm)	Circularity
C	1.82 ± 0.02	5.77 ± 0.07	0.6 ± 0.02
F	1.72 ± 0.04	4.91 ± 0.06	0.58 ± 0.02
H	1.82 ± 0.04	4.92 ± 0.07	0.73 ± 0.03

The effect of the treatments was not significant (*p* > 0.05) according to ANOVA analysis as compared with control.

## Data Availability

Not applicable.

## Sample Availability

Samples of the compounds are not available from the authors.
